# Supplementary material for: Multi-hierarchical profiling the structure-activity relationships of engineered nanomaterials at nano-bio interfaces
Source: Nat Commun. 2018 Oct 24;9:4416. doi: 10.1038/s41467-018-06869-9 (PMC6200803; doi:10.1038/s41467-018-06869-9)
Supplement: Supplementary file 1 — Supplementary Information [file 41467_2018_6869_MOESM1_ESM.pdf]

Supplementary Information

Multi-hierarchical Profiling the Structure-Activity Relationships of Engineered Nanomaterials at Nano-Bio Interfaces

Cai et al

Figures

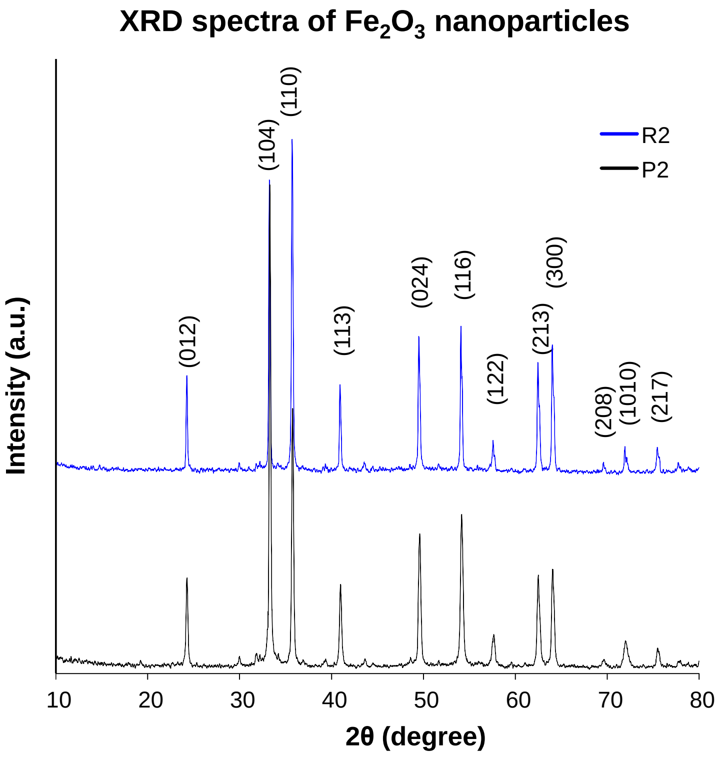

Supplementary Figure 1. XRD characterization of Fe2O3 nanoparticles

A Philips X'Pert Pro diffractometer equipped with CuKr radiation were used to obtain the XRD spectra of selected nanoplates (P2) and nanorods (R2).

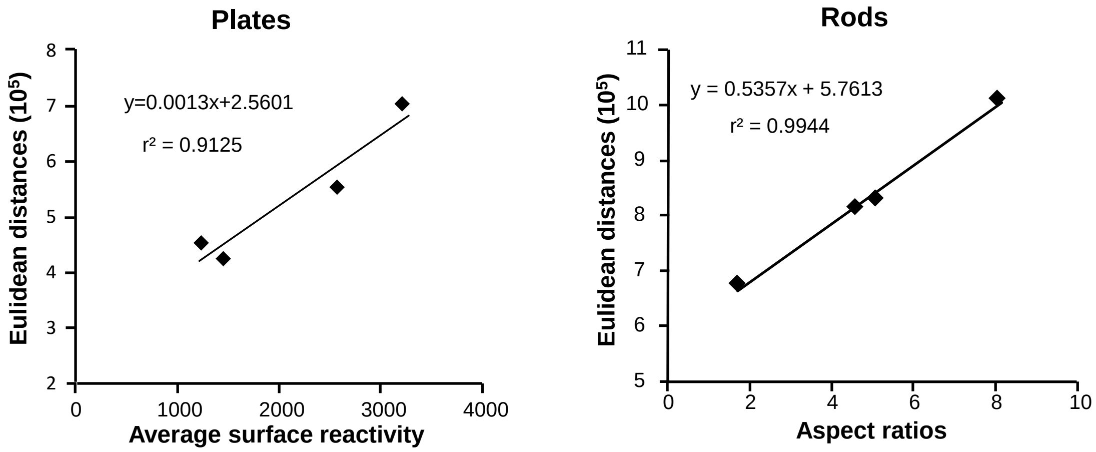

Supplementary Figure 2. Regression analysis of EDs and particle properties

THP-1 cells exposed to 100 µg/mL Fe_2O_3 nanoparticles were harvested and lyzed for metabolite extraction and nontargeted LC-MS analysis. The EDs represent the influences of each Fe_2O_3 nanoparticles to the global metabolite homeostasis in THP-1 cells. The r^2 values in regression analysis were used to estimate the relationships among variables.

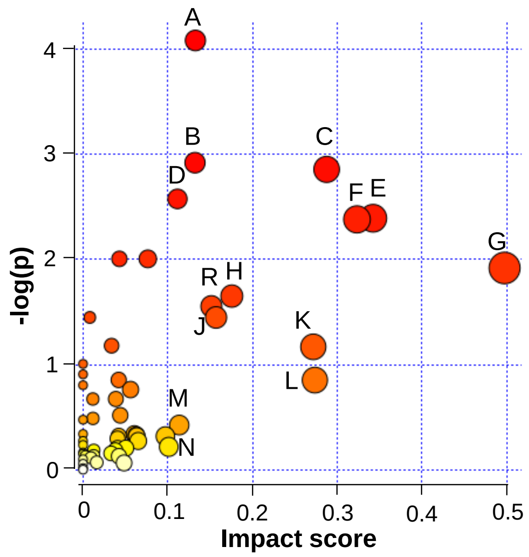

Supplementary Figure 3. Metabolite pathway analysis of putative metabolites

HMDB IDs of the 417 potential metabolites were input into the MetaboAnalyst for metabolite pathway analysis. We identified 14 pathways including sphingolipid metabolism (A), tryptophan metabolism (B), phenylalanine metabolism (C), pyrimidine metabolism (D), glycerophospholipid metabolism (E), beta-alanine metabolism (F), D-glutamine and D-glutamate metabolism (G), tyrosine metabolism (H), purine metabolism (I), pantothenate and CoA biosynthesis (J), sulfur metabolism (K), glutathione metabolism (L), propanoate metabolism (M) and primary bile acid biosynthesis (N). Pathway library for Homo sapiens was selected for the analysis. Hypergeometric test was performed in the over representation analysis. Relative-betweeness centrality algorithm was used in the pathway topology analysis.

A

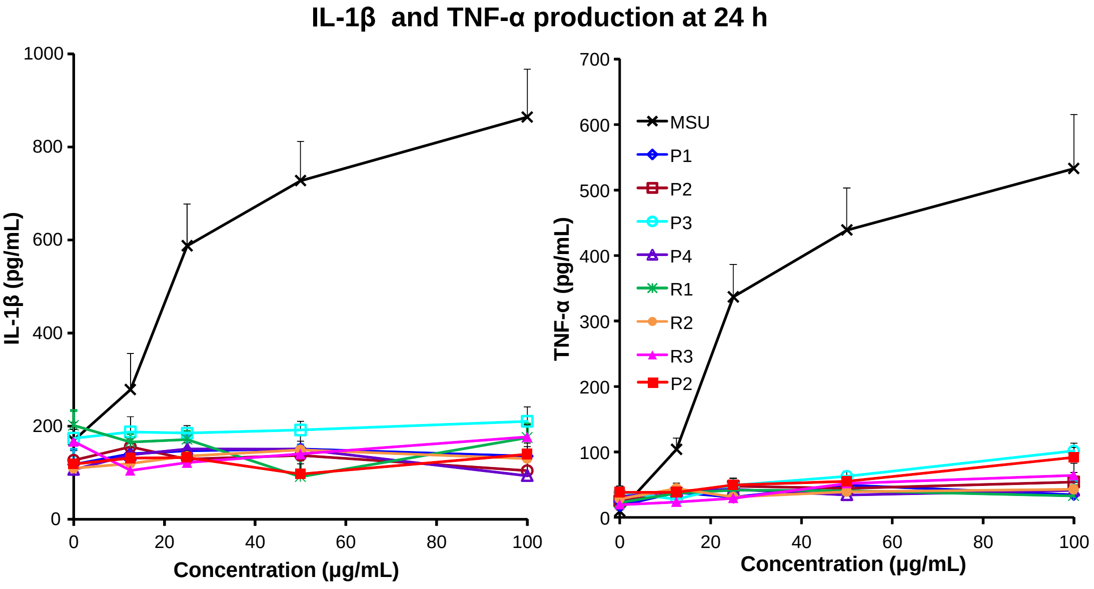

B

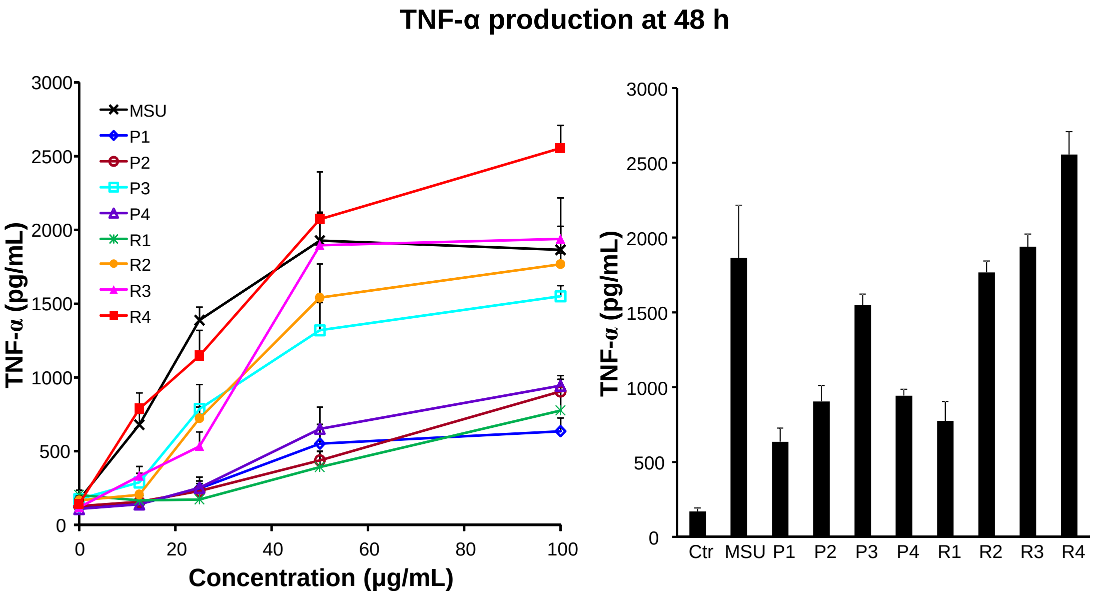

Supplementay Figure 4. Cytokine release in Fe_2O_3-treated cells

A) IL-1β and TNF-α production in THP-1 cells exposed to Fe_2O_3 for 24 h, and **B**) TNF-α production in THP-1 cells exposed to Fe_2O_3 nanoparticles for 48 h. After exposure to 0-100 μg/mL Fe_2O_3 nanoparticles for 24 or 48 h, the supernatants were collected to determine IL-1β and TNF-α productions. Data are shown as mean ± s.d. from 3 independent replicates.

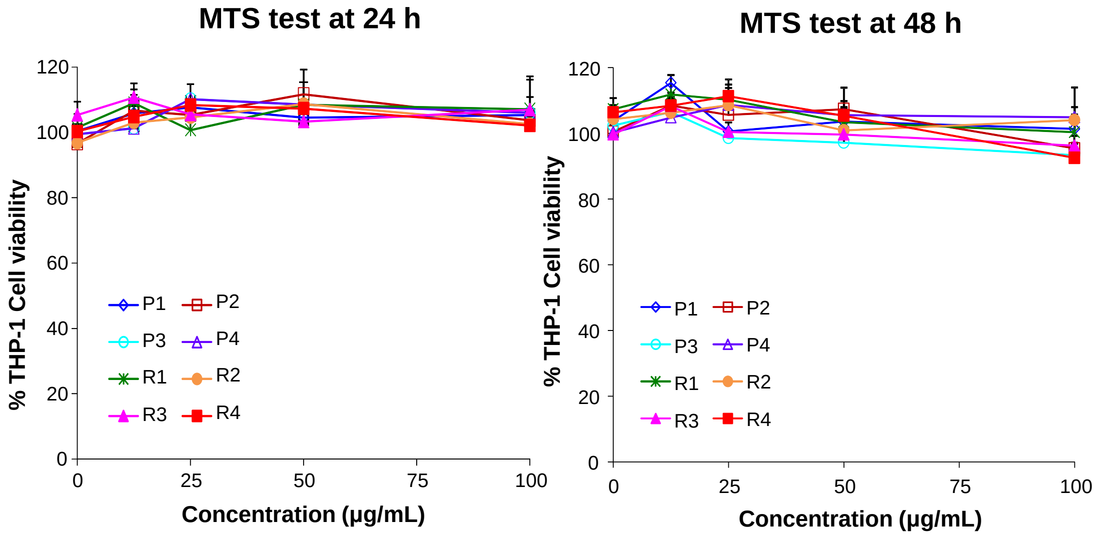

Supplementary Figure 5. Cytotoxicity assay

After exposure to 0-100 μg/mL Fe_2O_3 nanoparticles for 24 or 48 h, the MTS assay solutions were added to evaluate cell viabilities by measuring the absorbance on a SpectraMax M5 microplate spectrophotometer. Data are shown as mean ± s.d. from 4 independent replicates.

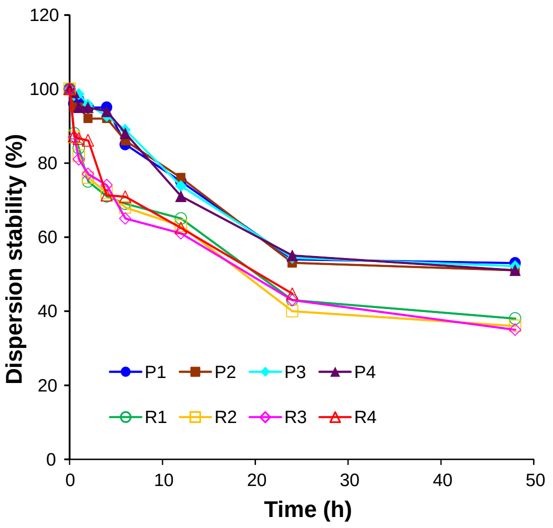

Supplementary Figure 6. Dispersion stability of Fe_2O_3 nanoparticles

Fe_2O_3 nanoparticles were dispersed in RPMI 1640 media at 25 μg/mL by probe sonication. The absorbance of tube suspensions was measured at 0, 0.5, 1, 2, 4, 6, 12, 24 and 48 h on a UV-Vis spectrometer at 550 nm. The suspension stability index could be calculated by a previously reported formula: $\frac{A0-Ai}{A0} \times 100\%$, where A0 is the original absorbance of Fe_2O_3 suspensions, while Ai represents the absorbance at various timepoints.

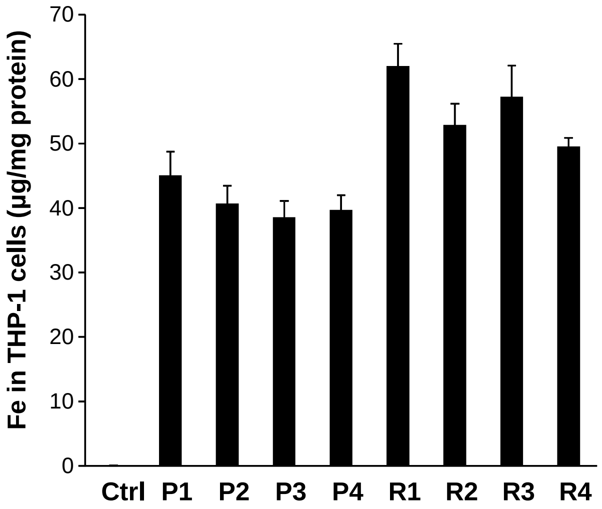

Supplementary Figure 7. Cellular uptake of Fe_2O_3 nanoparticles

THP-1 cells were treated with 25 µg/mL Fe_2O_3 nanoparticles for 6 h, washed with PBS three times, and collected by centrifugation. The cellular uptake levels of Fe_2O_3 nanoparticles in cell lysis solutions were determined by ICP-OES. The protein concentrations were measured by a Bradford assay. Data are shown as mean ± s.d. from 3 independent replicates.

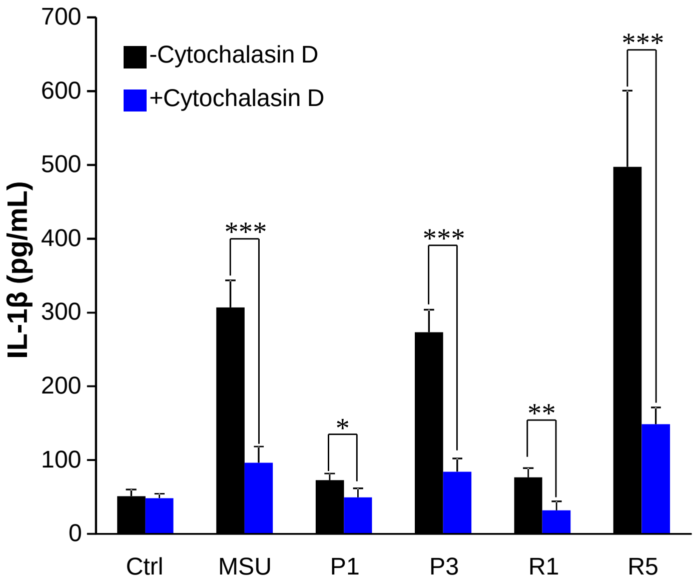

Supplementary Figure 8. Impacts of cytochalasin D in IL-1β production

To investigate the role of endocytosis in Fe_2O_3-induced IL-1β production, THP-1 cells were pretreated with an endocytosis inhibitor, cytochalasin D at 10 μg/mL for 3 h and then exposed to 100 μg/mL Fe_2O_3 nanoparticles for 30 h to determine IL-1β in supernatants. Data are shown as mean ± s.d. from 4 independent replicates. *p< 0.05, **p< 0.01, and ***p< 0.001 (two-tailed Student's t-test).

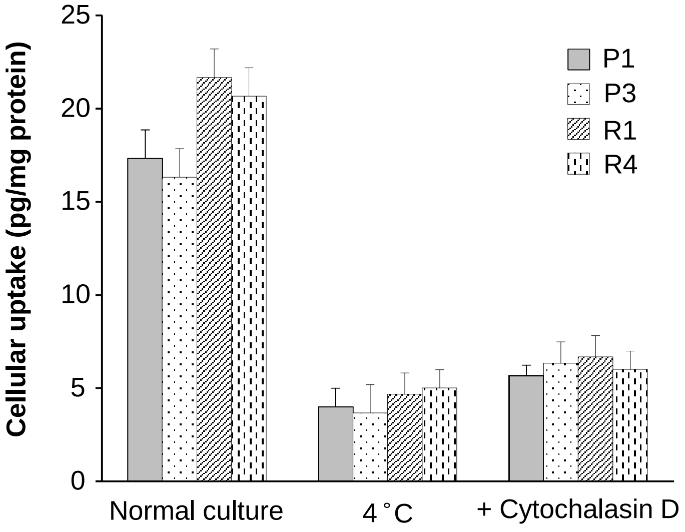

Supplementary Figure 9. Cellular uptake mechanism analysis

Four Fe_2O_3 nanoparticles including P1, P3, R1 and R4 were exposed to THP-1 cells cultured at 4 °C, 37 °C or cytochalasin D pretreated (10 μg/mL, 3 h) THP-1 cells. After 4 h incubation, the cells were rinsed with cold PBS and collected by centrifugation for cell digestion. The iron contents in cell lyses were measured by ICP-OES. Data are shown as mean ± s.d. from 3 independent replicates.

A

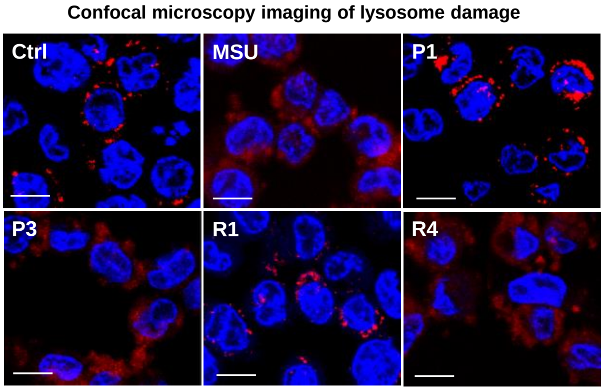

B

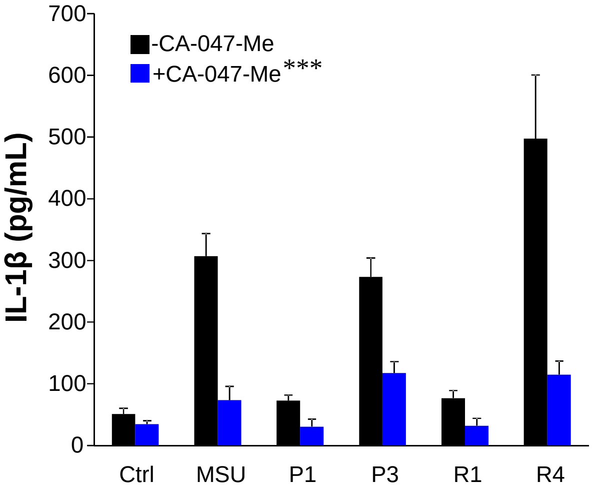

Supplementary Figure 10. Lysosome damage and cathepsin B release

A) Confocal microscopy imaging of cathepsin B release. THP-1 cells were seeded into 8-well chamber slides and incubated with Fe_2O_3 nanoplates (P1, P3) and nanorods (R1, R4) at 100 μg/mL for 16 h, and incubated with Magic Red for 30 min. After fixation, cells were stained with Hoechst 33342 dye, followed by visualization under a confocal 1P/FCS inverted microscope. Monosodium urate (MSU) crystals were used as the positive control. Scale bar is 10 μm. **B)** Influences of cathespsin B inhibitor in Fe_2O_3-induced IL-1β production. THP-1 cells were pretreated with a cathepsin B inhibitor, CA-074-Me at 10 μM for 3 h and then exposed to 100 μg/mL Fe_2O_3 nanoparticles for 30 h to determine IL-1β in supernatants. Data are shown as mean ± s.d. from 4 independent replicates. ***p< 0.001 compared to particle-treated cells without CA-047-Me treatment (two-tailed Student's t-test).

Tables

Supplementary Table 1. Squared correlation coefficients in regression analysis

	Diameter/ length	Thickness	Aspect ratio	Surface area	Zeta potential		Hydrodynamic size		Surface reactivity
					DI H_2O	RPMI1640	DI H_2O	RPMI1640	
**Plates**	0.00003	0.1322	0.0006	0.1076	0.2698	0.017	0.0773	0.102	0.9125
**Rods**	0.8056	0.1271	0.9944	0.0984	0.0267	0.0996	0.5615	0.4338	0.1206

Supplementary Table 2. Metabolite pathway analysis result from MetaboAnalyst

Pathway name	Total	Expected	Hits	Raw p	-log(p)	Impact
Sphingolipid metabolism	25	1.56	5	0.02	4.08	0.13
Tryptophan metabolism	79	4.92	9	0.05	2.92	0.13
Phenylalanine metabolism	45	2.80	6	0.06	2.86	0.29
Pyrimidine metabolism	60	3.74	7	0.08	2.58	0.11
Glycerophospholipid metabolism	39	2.43	5	0.09	2.39	0.34
beta-Alanine metabolism	28	1.74	4	0.09	2.38	0.32
D-Glutamine and D-glutamate metabolism	11	0.69	2	0.15	1.92	0.50
Tyrosine metabolism	76	4.74	7	0.19	1.65	0.18
Purine metabolism	92	5.73	8	0.21	1.55	0.15
Pantothenate and CoA biosynthesis	27	1.68	3	0.23	1.45	0.16
Sulfur metabolism	18	1.12	2	0.31	1.17	0.27
Glutathione metabolism	38	2.37	3	0.43	0.85	0.27
Propanoate metabolism	35	2.18	2	0.65	0.43	0.11
Primary bile acid biosynthesis	47	2.93	2	0.80	0.22	0.10

A 'Total' is the total number of compounds in the pathway; the 'Hits' is the actually matched number from the user uploaded data; the 'Raw p' is the original p value calculated from the enrichment analysis; the 'Impact' is the pathway impact value calculated from pathway topology analysis.
