# Supplementary material for: Multi-hierarchical profiling the structure-activity relationships of engineered nanomaterials at nano-bio interfaces
Source: Nat Commun. 2018 Oct 24;9:4416. doi: 10.1038/s41467-018-06869-9 (PMC6200803; doi:10.1038/s41467-018-06869-9)
Supplement: Supplementary file 4 — Description of Additional Supplementary Files [file 41467_2018_6869_MOESM4_ESM.docx]

Supplementary Data 1: Detailed information of the putatively identified metabolites and biological process related to their metabolism pathways
